# A Systematic Review on Artificial Liver for Implantation

**DOI:** 10.3390/jfb17020073

**Published:** 2026-02-02

**Authors:** Thi Huong Le, Kinam Hyun, Nima Tabatabaei Rezaei, Chanh Trung Nguyen, Sandra Jessica Hlabano, Van Phu Le, Keekyoung Kim, Kyo-in Koo

**Affiliations:** 1 Department of Electrical, Electronic and Computer Engineering, University of Ulsan, Ulsan 44610, Republic of Korea; lehuong94alhp@gmail.com (T.H.L.); nctrung1407@gmail.com (C.T.N.); sandrajessiehlabano@gmail.com (S.J.H.); levbk03@gmail.com (V.P.L.); 2 Department of Mechanical and Manufacturing Engineering, Schulich School of Engineering, University of Calgary, Calgary, AB T2N 1N4, Canada; kinam.hyun@ucalgary.ca (K.H.); nima.tabatabaeirezae@ucalgary.ca (N.T.R.); 3 Department of Medicine, Cumming School of Medicine, University of Calgary, Calgary, AB T2N 1N4, Canada; 4 Snyder Institute for Chronic Diseases, University of Calgary, Calgary, AB T2N 1N4, Canada; 5 Department of Biomedical Engineering, Schulich School of Engineering, University of Calgary, Calgary, AB T2N 1N4, Canada; 6 Basic-Clinical Convergence Research Institute, University of Ulsan, Ulsan 44610, Republic of Korea

**Keywords:** implantable artificial liver, vascularization, volumetric biofabrication

## Abstract

Chronic liver disease remains a leading cause of global mortality, yet organ shortages and transplant complications limit the efficacy of orthotopic liver transplantation. While extracorporeal support systems serve as temporary bridges, they fail to restore long-term patient autonomy or replicate complex biosynthetic functions. This systematic review, conducted in accordance with PRISMA 2020 guidelines, evaluates recent advancements in implantable artificial livers (IALs) designed for permanent functional integration. We analyzed 71 eligible studies, assessing cellular sources, fabrication strategies, maturation processes, and functional readiness. Our findings indicate significant progress in stem-cell-derived hepatocytes and bioactive scaffolds, such as decellularized extracellular matrix (dECM). However, a critical technological gap remains in scaling current sub-centimeter prototypes toward clinically relevant volumes (~200 mL). Key engineering challenges include integrating hierarchical vascular networks, requiring primary vessels exceeding 2 mm in diameter for surgical anastomosis, and functional biliary systems to prevent cholestatic injury. Furthermore, while micro-vascularization and protein synthesis are well documented, higher-order functions such as spatial zonation and coordinated metabolic stability remain underreported. Future clinical translation necessitates advancements in multi-cellular patterning, microfluidic-driven maturation, and autologous reprogramming. This review provides a comprehensive roadmap for bridging the gap between biofabricated constructs and organ-scale hepatic replacement, emphasizing the need for standardized functional benchmarks to ensure long-term success.

## 1. Introduction

Liver disease causes approximately 2 million annual deaths, accounting for 3.5% of global mortality [1]. While orthotopic liver transplantation (OLT) remains the definitive treatment for end-stage failure, its application is severely limited by a persistent organ shortage; currently, less than 10% of global transplant requirements are met [2]. Furthermore, OLT involves significant long-term risks. Acute rejection affects 15% to 25% of recipients within the first year [3], necessitating lifelong immunosuppression. These regimens increase susceptibility to opportunistic infections and chronic kidney disease, which affects 20% of patients within five years [4]. Additionally, stringent eligibility criteria exclude many patients with comorbidities or advanced age. These constraints underscore the urgent need for alternative therapeutic strategies beyond conventional human organ replacement.

Over the past few decades, extracorporeal liver support systems have been developed to serve as a clinical “bridge,” stabilizing patients until they can undergo orthotopic liver transplantation or achieve spontaneous hepatic recovery [5]. These systems are broadly categorized into non-biological artificial liver (AL) and cell-based bioartificial liver (BAL) types. AL devices, such as the Molecular Adsorbents Recirculation System (MARS), rely primarily on physical and chemical detoxification mechanisms, including dialysis and adsorption, to remove albumin-bound toxins. While MARS has been shown to significantly reduce serum bilirubin levels, large-scale clinical trials, such as the RELIEF trial, have demonstrated no significant improvement in 28-day survival rates compared with standard medical therapy (60.7% vs. 58.9%, *p* = 0.74) [5]. This underscores the critical limitation of AL: it lacks the essential biosynthetic and metabolic functions of hepatocytes, such as albumin synthesis and ammonia detoxification.

To address this, BAL systems integrate bioreactors containing high densities of functional hepatocytes, typically ranging from 1 × 10^9^ to 4 × 10^10^ cells [5], to replicate complex liver functions. However, even advanced BAL systems, such as the Extracorporeal Liver Assist Device (ELAD) failed to meet primary endpoints in major clinical trials. A primary concern with ELAD is its reliance on the C3A cell line, a clonal derivative of human hepatoblastoma-derived HepG2 cells [6]. While these cells offer practical advantages in terms of robust proliferation and scalability, they exhibit significant metabolic deficiencies compared to primary human hepatocytes, including impaired ureogenesis and lower cytochrome P450 (CYP450) activity [6]. Consequently, the VTI-208 trial showed no statistically significant difference in survival for patients with alcohol-induced liver decompensation (51.0% for ELAD vs. 49.4% for control) [6]. Beyond these efficacy and biological gaps, extracorporeal systems remain highly restrictive; they are hospital-bound, often requiring 6 to 8 h of treatment per session [7], which severely impairs patient autonomy and fails to restore a sustainable long-term quality of life.

Thanks to recent breakthroughs in biofabrication [8,9,10,11,12,13,14,15] and stem cell biology, there has been a shift in the research paradigm toward implantable artificial livers. Unlike extracorporeal systems, these constructs are designed as biofabricated tissues or organs capable of sustained in vivo implantation and functional integration. A primary driver of this transition is the advancement of induced pluripotent stem cell (iPSC) technology, which now enables the generation of hepatocyte-like cells (HLCs) with good metabolic function. Furthermore, various biofabrication techniques, including 3D bioprinting, have enabled the fabrication of hepatic architectures to mimic the high cellularity of the native liver. Despite this rapid technological progress, the terminology distinguishing these implantable constructs from extracorporeal BALs remains ambiguous. Due to the multidisciplinary nature of this field, involving tissue engineering, materials science, and regenerative medicine, various terms such as “bioengineered liver”, “tissue-engineered liver graft”, and “implantable hepatic construct” have been utilized inconsistently. In this article, the term “artificial liver for implantation” or “implantable artificial liver (IAL)” is used to refer to a construct intended for long-term or permanent in vivo implantation, distinct from extracorporeal AL/BAL support systems that serve only as a temporary bridge to human liver transplantation. Despite the proliferation of literature on hepatic tissue engineering, there remains a lack of systematic evaluation focusing specifically on the transition from bench-top micro-models to clinically viable, organ-scale implants.

The core concept of this review is to evaluate recent IAL advancements through the lens of ‘clinical readiness.’ To effectively replace a diseased liver, an IAL must reach a clinically relevant scale; for instance, a minimum volume of approximately 200 mL is required for liver transplantation in pediatric patients [16,17]. However, a significant gap remains between these requirements and current technology, as one of the most biofabricated prototypes reported in 2025 is 1 cm^3^ (10 mm × 10 mm × 10 mm) [18]. Beyond scalability, the survival of such large-scale constructs depends on preventing core necrosis, which necessitates a hierarchical vascular architecture. This includes a dense micro-vascular network for nutrient diffusion and at least one primary vessel with a diameter exceeding 2 mm to allow vascular surgical anastomosis to the host’s circulatory system [19]. Furthermore, the integration of a functional biliary system is another critical requirement to prevent cholestatic injury and ensure long-term graft maturation. To promote biocompatibility and minimize the risk of immune rejection, recent research prioritizes the use of stem-cell-derived hepatic cells and biodegradable materials that support in vivo tissue remodeling. By synthesizing these technical requirements, this review paper aims to summarize recent advances in IALs, emphasizing their cellular composition, material and fabrication strategies, vascular and biliary architecture, maturation processes, volumetric scalability, and functional validation both in vitro and in vivo, following the preferred reporting items for systematic reviews and meta-analysis PRISMA guidelines [20] to provide a rigorous assessment of the current state-of-the-art and the remaining hurdles toward clinical translation.

## 2. Methods

This review was conducted in accordance with the PRISMA 2020 statement [20]. The protocol was previously registered on protocols.io (https://doi.org/10.17504/protocols.io.n2bvj1rw5vk5/v1 accessed on 28 January 2026). A comprehensive literature search was performed across three electronic databases: Web of Science, PubMed, and Scopus. The search strategy utilized combinations of keywords, including ‘artificial liver’, ‘liver tissue engineering’, and ‘engineered liver’. Search results were restricted to original research articles published in English since 2015. Document types such as review articles, meta-analyses, conference abstracts, and proceedings were excluded. The specific search strings employed for each database are detailed in Table 1.

The initial search yielded 1422 records (Web of Science: 464, PubMed: 379, Scopus: 579). After removing 780 duplicates, 642 unique titles and abstracts remained for screening. Six independent authors screened these records and excluded 571 articles based on the following criteria: (a) extracorporeal artificial liver support systems; (b) liver-on-a-chip models; (c) cell aggregates or droplets lacking strategies for tissue integration; and (d) cell-free constructs seeded with cells post-fabrication.

**Figure 1:**
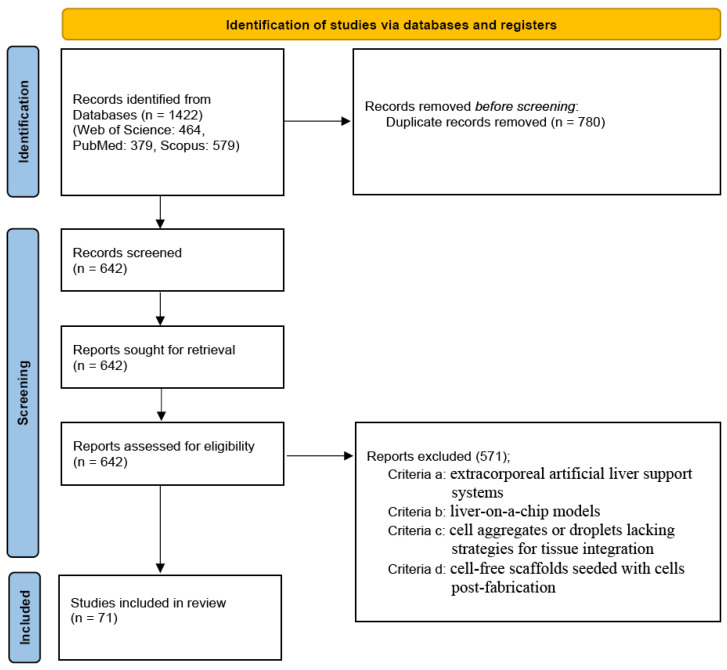
The PRISMA flow diagram shows the study selection process.

Specifically, exclusion criterion (c) was further refined to exclude: (c-1) stem cell aggregates without evaluation of vascularization, and (c-2) hepatic cell aggregates lacking endothelial cell co-culture. Similarly, criterion (d) was specified to exclude: (d-1) stem cell seeding without vascularization analysis, and (d-2) hepatic cell seeding without endothelial cell integration. Ultimately, 71 eligible studies were included in this review. All the excluded studies were independently verified by another author. The study selection process is illustrated in the PRISMA flow diagram (Figure 1).

## 3. Results and Discussion

### 3.1. Cells

#### 3.1.1. Cell Source

According to the studies analyzed in this review, as shown in Table 2, human or rat tissues were predominantly used as sources for deriving cells. This is distinctly indicative of the diverse experimental objectives aimed at making profound advancements in the field of hepatic tissue engineering, as well as the approaches implemented for biofabrication [14,21,22,23,24,25,26]. Cells derived from human sources were primarily preferred for in vitro studies due to their superior physiological properties as well as their meticulous alignment with human native liver metabolic functionality and the potential to mimic native extracellular matrix (ECM) environment [25,27,28,29,30,31,32]. These model systems are exceptionally well-suited for studies that require demonstrating targets in disease models, drug and detoxification, and possible clinical applications. Conversely, rat-derived cells were commonly employed in research that rather focused on construct development, studies investigating perfusion in artificial constructs, optimization coupled with proving or disproving literature concepts as a basis for further studies due to their easy accessibility and availability, reproducibility, and isolation protocols that have already been successfully ascertained [26,33,34,35]. Human cell sources offer clinical relevance and metabolic fidelity, while rat cells provide practical advantages for optimization and proof-of-concept studies.

#### 3.1.2. Cell Type and Cell Line

Cell type refers to the biological and functional classification of cells. Among the reviewed studies, hepatocytes were the dominant functional cell type employed, as described in Table 2. As liver parenchymal cells, they were used as primary hepatocytes, HLCs, or derived hepatocytes due to their essential role in liver-specific functions, including metabolism, detoxification, albumin and bile biosynthesis, and overall maintenance of ECM composition [26,33,43,44,55,59,60,61,62,87]. In addition, as illustrated in Table 2, several non-parenchymal liver cell types, including liver sinusoidal endothelial cells, stellate cells, macrophages, fibroblasts, and biliary epithelial cells, were incorporated into experimental model systems. This inclusion was prompted by the need to promote vascularization and angiogenesis, enhance elasticity and regenerative properties, support the remodeling of ECM composition, and facilitate the formation of the functional biliary networks [32,33,36,45,46,56,62,63,67,87]. Support cells such as mesenchymal stem cells (MSCs), iPSCs and progenitor cells were also frequently included in studies because of their ability to differentiate into hepatic cells [34,44,47,48,71,72,83,90]. These cells demonstrated increased regenerative capacity, decreased cell death over time, and improved long-term stability of artificial constructs.As presented in Table 3, the majority of studies employed two- or multi-cell-type co-culture systems to replicate the complex, heterogeneous cellular environment of native liver tissue, typically combining hepatocytes with non-parenchymal or support cell types.

It is, however, important to note that some studies used cell lines rather than primary cell types. Common examples used across evaluated studies included HepG2, HepaRG, C166 and C3A cells [14,23,24,28,36,42,54,57,64,82]. While cell lines are beneficial for model system optimization studies due to their stability and easy maintenance, they recurrently exhibit altered metabolic activity, simplified gene expression of mRNA, and distorted detoxification activity in comparison to primary cell types [37,54] necessitating validation with primary hepatocytes in future studies.

Primary cell types provide superior physiological relevance to the native microenvironment of the liver, while cell lines offer practical benefits for reproducibility, scalability and optimization.

#### 3.1.3. Cell Density

Cell density is a critical parameter influencing cell viability, functional maturation, and nutrient diffusion in liver biofabrication [33,37,57,64]. The reported cell densities varied depending on the architecture of constructs, the composition of hydrogel used, and conditions and methods for cell culturing but were generally encompassed within the following density ranges;

- 0.5 × 10^6^–1 × 10^7^ cells/mL in 3D hydrogels and constructs that employed dECM [14,21,38,49,70]
- 4 × 10^3^–1 × 10^5^ cells/cm^2^ in 2D or membrane-supported cultures [56,58,65,90]

Higher hepatocyte densities correlated with improved albumin secretion, urea synthesis, and CYP450 activity, particularly in perfusion and vascularization-focused studies [14,33,37,50,64]. However, excessively high densities caused diffusion limitations and hypoxia in the cell culture environment, highlighting the need for optimized microchannel architecture and perfusion strategies [52,64]. Optimal cell density is essential for balancing functionality and nutrient transport in engineered liver constructs.

### 3.2. Materials and Method for IAL Fabrication

Within the field of biofabrication, the term ‘scaffold’ typically refers to a structural framework or hydrogel matrix that serves as a synthetic ECM scaffold, providing physical support for encapsulated cells as they mature into functional tissue. However, recent advancements have introduced scaffold-free strategies, leveraging autonomous cellular self-aggregation and organoid formation, which bypass the need for exogenous materials. Consequently, in this manuscript, the broader term ‘construct’ is adopted to describe any artificially fabricated hepatic architecture, encompassing both scaffold-based and scaffold-free systems in their pre-maturation.

#### 3.2.1. Construct Composition

Construct materials play a central role in liver tissue engineering, as they are required to recapitulate key structural, mechanical, and biochemical features of the native hepatic ECM. An appropriate construct provides not only mechanical support but also essential microenvironmental cues that govern hepatocyte adhesion, survival, proliferation, and functional maintenance [24,72]. Given the anchorage-dependent nature of hepatocytes and their pronounced sensitivity to changes in the surrounding matrix, preserving liver-specific functions remains a major challenge following cell isolation and in vitro culture.

Consequently, considerable research efforts have been devoted to developing construct materials that ensure adequate mass transport, mechanical integrity, biocompatibility, controlled biodegradation, and bioactivity. To address these requirements, a wide range of materials and fabrication strategies have been explored, and can generally be classified into natural hydrogels, synthetic polymers, and dECMs for the construction of 3D artificial liver constructs, as described in Table 4.

##### Nature Hydrogel

Natural hydrogels have been widely explored as construct materials for artificial liver tissue engineering because of their intrinsic biocompatibility and close resemblance to the native ECM. Alginate is among the most used natural polymers and is typically crosslinked via ionic interactions with divalent cations such as Ca^2+^ or Ba^2+^. Owing to its excellent printability and rapid gelation, alginate is particularly suitable for extrusion-based 3D bioprinting and has been employed in multi-material bioprinting systems and hepatic plate–mimetic liver models. Snyder et al. reported that alginate enables the development of an asynchronous multi-material bioprinting (SMMB) system with improved printing resolution and the ability to organize multiple cell types into heterotypic arrays before deposition [24]. The resulting constructs showed increased cell size and metabolic activity, indicating enhanced drug uptake capacity. However, alginate exhibits relatively slow and poorly controllable degradation, which can limit cell migration and often necessitates chemical modification or blending with bioactive components to enhance cell–matrix interactions [54].

Gelatin and gelatin methacryloyl (GelMA) are widely used protein-based hydrogels due to their favorable bioactivity and ease of crosslinking. Gelatin undergoes thermal gelation, whereas GelMA can be photo-crosslinked via methacrylate groups, enabling improved shape fidelity and printability. Several reports have shown that gelatin supports good cell attachment and spreading and maintains hepatic-specific functions in bioprinted liver construct with enhanced metabolic activity and post-transplantation vascularization [68,70]. Nevertheless, gelatin-based hydrogels suffer from limited mechanical stability, as they tend to melt or degrade rapidly at physiological temperature (37 °C), and GelMA often requires reinforcement or composite strategies to achieve sufficient mechanical strength for long-term culture.

Among all natural polymers, collagen is the primary structural protein of the native liver ECM, providing excellent bioactivity and supporting robust cell migration, polarization, and tissue remodeling through thermal self-assembly. Collagen-based constructs have been extensively utilized in perfused liver models, microsystems, and angiogenic liver constructs due to their superior biological performance. Yang et al. encapsulated human hepatocytes in collagen-based microbeads with MSCs and cultured them in a 3D-printed tubular perfusion bioreactor, enhancing oxygen and nutrient diffusion to the cells [45]. That novel perfusion system sustained the survival and function of human liver cells within a centimeter-sized 3D construct for up to 30 days. Liu et al. developed a liver microsystem to enhance nutrient transport for the high-density culture of hepatocytes [37]. However, due to their weak mechanical strength and slow gelation kinetics, collagen hydrogels are often combined with other polymers (e.g., alginate, heparin) or chemically crosslinked to improve structural stability and handling properties [34,37].

Fibrin, formed through enzymatic crosslinking of fibrinogen by thrombin, is another biologically active hydrogel frequently used in liver tissue engineering. Not only do fibrin matrices promote excellent cell migration and angiogenesis, but they are also well-suited for cell encapsulation and grafted liver tissue formation [31,69]. Despite these advantages, fibrin undergoes rapid biodegradation under physiological conditions, limiting its ability to maintain long-term structural integrity.

##### Synthetic Polymer

Synthetic polymers have been extensively investigated for fabrication of artificial liver constructs due to their well-defined chemistry, tunable mechanical properties, and excellent printability. Poly (ethylene glycol) (PEG) and its photo-crosslinkable derivative PEG-diacrylate (PEGDA) are used for developing a 3D co-culture construct of hepatocytes with non-parenchymal ECs and pericytes. Co-culture promotes the formation of robust microvascular tubules, preserves hepatocyte phenotype, and enhances liver-specific functions, including albumin production and CYP450 activity [33,84]. On the other hand, Pang et al. employed biodegradable poly-L-lactic acid to fabricate biodegradable fiber for culturing HepG2 and TMNK-1 cells in a perfusion system. The system enabled increased hepatic function and well-maintained cell viability, demonstrating the importance of an independent medium flow supply for cell growth and function provided by the current 3D construct [29]. Another study presented an approach in which modified polyethersulfone (PES) hollow fiber membranes (HF) were used to create a human liver system under static and dynamic conditions. The membrane surface supported cell attachment and self-assembly into tissue-like structures. Sinusoidal cells formed tube-like networks around hepatocyte cords, promoted by stellate cells. The system maintained albumin and urea production for up to 28 days, preserved drug biotransformation activity, and sustained physiologically relevant oxygen levels [14]. Although synthetic materials demonstrate excellent printability through photo- or radiation-based crosslinking, their lack of intrinsic bioactivity, results in poor cell adhesion, often requiring surface functionalization or incorporation of bioactive ligands [29,64].

dECM

Current in vitro fabrication techniques are unable to fully recreate the complex biochemical components and unique physical properties of native liver ECM, including topography, pore size, fiber orientation, stiffness, elasticity, and hierarchical architecture. Consequently, dECM–based materials have gained increasing popularity as construct sources because they preserve the native ECM composition, ultrastructure, and, in some cases, the embedded microvascular networks of the original tissue. In this approach, donor cellular components are removed through perfusion-based decellularization using detergents. At the same time, the remaining ECM construct can be subsequently repopulated with desired cell types to regenerate functional tissue constructs [35,71,88].

Both porcine liver–derived dECM and rodent-derived liver dECM (rat and mouse) have been widely used in fundamental studies and disease modeling due to their biological relevance and accessibility. In 2022, Wu et al. demonstrated the generation of long-term vascularized hepatic parenchyma at ectopic sites using porcine decellularized liver constructs combined with stem cells [74]. They found that the hepatic grafts survived at ectopic sites, maintained hepatocyte-specific functions, and efficiently anastomosed with host vasculature. Notably, HLCs within the grafts expanded more than 9-fold over 4 weeks in immunocompetent rats with liver injury. Zhang et al. developed decellularized liver constructs from whole mouse livers and recellularized them with hepatic stem/progenitor cells for liver tissue engineering [86]. The constructs preserved native microstructure and ECM components, supporting efficient cell attachment and hepatic differentiation, which was superior to conventional two-dimensional culture. These engineered grafts formed hepatic-like tissue, produced functional hepatic proteins in vitro, and improved liver function and survival in a mouse model of liver cirrhosis after heterotopic transplantation.

Matrigel, a basement membrane extract enriched in laminin, collagen IV, entactin, and growth factors, is frequently used as a benchmark ECM material in liver tissue engineering. It provides excellent support for cell attachment, migration, and differentiation and recapitulates key aspects of the hepatic microenvironment [58,90]. Nevertheless, Matrigel suffers from poor structural and mechanical stability, and limited suitability for reproducible 3D construct fabrication, which significantly constrains its translational potential.

#### 3.2.2. Construct Structure and Its Fabrication

The structural design of liver constructs plays an important role in recapitulating native hepatic architecture, particularly with respect to vascularization and biliary organization. Across current liver tissue–engineering strategies, most constructs successfully incorporate microvascular networks, whereas the formation of large-diameter vessels (>1 mm) suitable for surgical anastomosis and functional bile ducts remains a significant challenge (Table 5).

##### Layered and Cell-Sheet–Based Technique

Layered and cell-sheet–based approaches consistently demonstrate the ability to generate microvascularized constructs after implantation. Engineered hepatocyte–fibroblast sheets and layer-by-layer (LbL) cell-sheet assemblies promote rapid host-derived vascular infiltration, enabling the formation of perfused microvascular networks in vivo without the need for pre-formed endothelial lumens or stem-cell–derived vasculature. Sasaki et al. introduced a platform for creating a vascularized liver tissue model using limited CPHs and co-culturing HUVECs/NHDFs via the layer-by-layer technique [34]. The LbL cell-coating technique significantly enhanced vitro liver function, as shown by increased human albumin production and CYP450 activity. After subcutaneous transplantation, the vascularized liver tissue exhibited higher early-stage albumin production than non-vascularized tissue or a hepatocyte suspension. Another study by Wang et al. fabricated 3D-imprinted cell-sheet technology to build biomimetic hepatic lobules [51]. Layer-by-layer imprinting enabled the formation of high-density hepatic lobules with intact cell interactions. Endothelial cell infiltration generated vascularized channels for nutrient and drug perfusion, allowing integration into a liver-on-a-chip for drug screening and assembly into larger constructs that promoted liver regeneration. However, despite robust microvascularization, such systems lack macroscale vessels (>1 mm) limiting their translational potential for large graft integration.

##### Spheroid/Organoid/Self-Assembling

Spheroid-, organoid-, and self-assembling systems emphasize cellular self-organization and paracrine-driven morphogenesis. Several studies report functional microvascular integration with host circulation following implantation, particularly when endothelial cell–containing spheroids are used. In 2024, Luo et al. proposed a straightforward and time-efficient approach to rapidly self-assemble mini-livers (RSALs) from primary hepatocytes within 12 h. RSALs formed large constructs (5.5 mm) with high viability and enhanced liver function, achieved functional vascularization within 2 weeks after mesenteric transplantation, and protected mice from 90% hepatectomy–induced liver failure, highlighting their bioartificial liver potential [69]. Nonetheless, vascularization in these models is typically emergent rather than architecturally controlled, and no studies report the presence of surgically anastomosable vessels [31,65]. Bile duct formation is also uncommon, highlighting the difficulty of reconstructing biliary features at the microscale.

##### Sacrificial Templating/Embedded Molds

Sacrificial templating, embedded molding, and decellularized construct strategies enable more precise spatial control over vascular fabrication. Re-endothelialized whole-organ or partial liver constructs preserve native vascular hierarchies, including large vessels exceeding 1 mm, making them uniquely suitable for vascular anastomosis. Hussen et al. demonstrated successful re-endothelialization of porcine liver constructs using heparin–gelatin vessel precoating, which enhanced endothelial cell attachment, migration, and anti-platelet properties. In vivo, the modified constructs showed no coagulation and preserved parenchymal cell function, highlighting their potential for developing human-sized engineered liver grafts with non-thrombogenic, patent vasculature for liver assist systems or transplantation [53]. Saleh et al. conjugated homogenized liver ECM into decellularized rat liver constructs to enhance their structural integrity and functional performance [41]. The results showed that the conjugated constructs have superior micro- and ultrastructural, as well as biochemical characteristics. Following intrahepatic transplantation, the conjugated constructs promoted greater recruitment of hepatic regenerative cells and improved angiogenesis, indicating their potential to enhance construct quality for liver transplantation. However, while the vascular tree is structurally preserved, most approaches do not achieve concurrent regeneration of a functional biliary network, unless bile duct structures are explicitly patterned or cholangiocyte populations are incorporated using advanced biofabrication techniques.

##### Constructs (Whole-Organ or Partial)

Construct-based systems, including whole-organ decellularized matrices and bioprinted constructs, represent a bridge between architectural fidelity and functional complexity. Many studies report microvascular networks and preserved large vessels, particularly in decellularized liver constructs and 3D-bioprinted liver models. Deng et al. constructed a 3D-bioprinted liver using In vivo–expanded primary hepatocytes with strong safety and functional robustness [50], or a study from Taymour’s group has succeeded in the fabrication of a complex in vitro triple-culture hepatic sinusoid model [21]. In these studies, they demonstrated the presence of blood vessels larger than 1mm, the cellular interactions occurring in the culture model enhanced the liver cell functions and extended survival in liver-injured mice. These constructs show improved angiogenesis, hepatocyte viability, and functional recovery in vivo. Furthermore, although bile duct reconstruction remains limited, several studies have demonstrated biliary tree formation through controlled co-culture or spatial patterning of hepatocytes and cholangiocytes. Research from Guo et al. demonstrated that co-culture generated hepatocyte–endothelial interfaces with better polarity, bile canaliculi formation, and enhanced liver functions compared to monocultures [46].

##### Microfluidic “Liver-on-Chip”/Perfused Bioreactors

Microfluidic liver-on-chip platforms and perfused bioreactors offer precise control over microscale fluid dynamics and sinusoid-like perfusion. These systems commonly incorporate capillary-sized channels that enhance mass transport and hepatocyte polarization, closely mimicking hepatic plates. While bile canaliculi and bile secretion pathways can be induced in vitro, these platforms lack both large-diameter vessels and clinical implantability, confining their application primarily to disease modeling, drug screening, and mechanistic studies [37,73]. However, recently there have also been a few studies on implantation, such as the study by Deng et al. A microfluidic platform enabled the generation of collagen microspheres encapsulating hESCs, which were efficiently differentiated into HLCs and self-assembled with endothelial cells to form dense pre-vascularized liver tissue. These constructs resembled native liver, successfully engrafted in the mouse liver, and showed improved hepatic function in vivo [44].

Vascularization is critical for functional engineered liver tissues. Most current systems achieve only microscale vascularization and lack the large vessels needed for building clinically relevant liver tissues. As a result, construct design must balance structural complexity with reproducibility and scalability. Micro-vascularization is widely achievable across platforms, whereas the integration of large vessels (>1 mm) is restricted mainly to decellularized or whole-organ–derived constructs. In contrast, bile duct formation is better reproduced in controlled in vitro systems but often lacks adequate vascular integration. Future construct designs must integrate hierarchical vasculature with spatially organized biliary networks to more fully recapitulate native liver structure and function.

#### 3.2.3. Construct Maturation

The maturation of artificial liver constructs represents a critical transitional phase during which fabricated structures achieve physiological stability, liver functionality, and long-term cellular viability. Without proper maturation, constructs often suffer from hypoxia, nutrient deficiency, and waste accumulation, especially in thicker or densely constructs. Following fabrication and cell seeding, constructs require controlled culture conditions that promote cellular organization, differentiation, metabolic activity, and extracellular matrix remodeling. Thus, the maturation process bridges the gap between structural assembly and biological performance, determining whether artificial tissue can effectively mimic native liver tissue. To address diffusion limitations and support functional hepatic tissue development, maturation strategies have been developed from static culture systems to dynamic perfusion-based platforms. These approaches differ in technological complexity, physiological relevance, and scalability. Selection of an appropriate maturation strategy depends on construct size, cell source, architectural complexity, and desired functional outcomes (Table 6).

Static culture systems involve the simple immersion of liver constructs in culture medium without active fluid movement. This method relies entirely on passive diffusion for nutrient delivery, oxygenation and waste removal. Due to their simplicity, cost-effectiveness, and compatibility with standard laboratory equipment, static systems are widely used in early-stage studies and high-throughput screening. However, limited mass transport restricts their applicability to thin constructs (<200 µm) or low cell densities. Nguyen et al. fabricated a 3D artificial liver using an extrusion bioprinting technique. The constructs were matured under static culture conditions for 10 days [14]. Gao et al. developed a bioactive hybrid construct composed of topographically modified polycaprolactone fibers. Cells were seeded onto the construct and incubated for up to 72 h [42].

**Figure 2:**
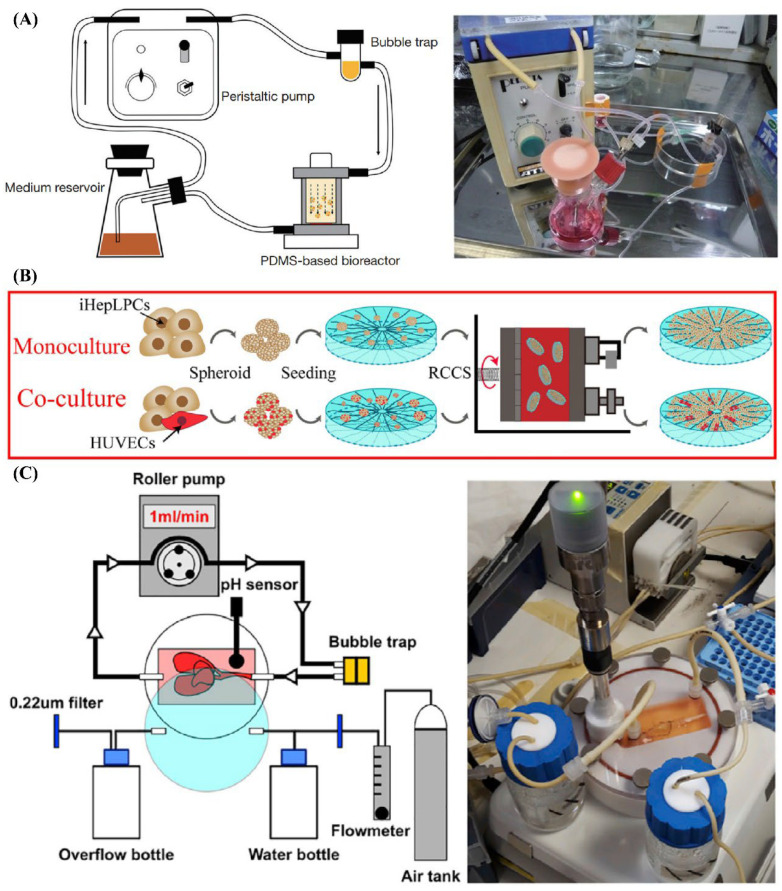
The Construct Maturation Approaches Using Dynamic Culture Systems. (**A**) Dynamic culture with perfusion chamber/system. Reprinted from Ref. [23], (**B**) Rotary culture system. Reprinted from Ref. [46], (**C**) Dynamic culture with bioartificial liver system. Reprinted from Ref. [25].

Dynamic culture systems introduce controlled medium flow to enhance mass transport and provide physiologically relevant mechanical cues. Featured dynamic culture systems are exhibited in Figure 2. Perfusion chamber systems utilize continuous or intermittent flow to supply nutrients and apply shear stress, significantly enhancing hepatocyte function and tissue viability in larger or more complex constructs. He et al. constructed highly dense, endothelialized hepatic cell aggregates using a microwell device. The aggregates were mixed with biodegradable poly-L-lactic acid fibers and cultured in a bioreactor under perfusion for 10 days [23]. Yang et al. encapsulated cells in collagen using a microfluidic device. The resulting beads were maintained within a 3D-printed channel and perfused with culture medium for up to 30 days [45].

Microfluidic devices represent an advanced subset of dynamic culture systems, offering precise control over microscale flow and enabling sinusoid-like perfusion patterns. Although typically limited to small volumes, these platforms are particularly valuable for liver-on-chip models, drug screening, and mechanistic studies. Liu et al. designed a liver microsystem to culture high-density hepatocytes in a 3D large-scale for 7 days. The experimental platform included 20 mL of culture medium, a peristaltic pump, a gas-exchange chip, and a liver-on-chip device as the core microsystem [37].

Rotary culture systems provide a low-shear, continuously mixed environment that promotes uniform aggregate formation, prevents sedimentation, and maintains 3D organization. This approach is especially effective for generating large numbers of spherical microtissues that can serve as modular building blocks for larger constructions. Guo et al. fabricated silk fibroin–collagen I composite constructs. A rotary cell culture system was applied to culture and mature the cell–construct constructs for up to 3 weeks [46].

At larger scales, bioartificial liver (BAL) systems represent advanced extracorporeal perfusion platforms designed to support long-term maturation of large tissue constructs, particularly in translational and clinical contexts. These systems integrate engineered hepatic tissues with controlled perfusion circuits that temporarily replace or supplement native liver function. Lorvellec et al. generated and tested an in vitro whole-organ “bioreactor-grown artificial liver model” over 27 days. This system consisted of a custom-designed bioreactor enabling long-term 3D culture of human induced pluripotent stem cell–derived HLCs within a mouse decellularized liver construct [25].

Hybrid maturation strategies combine static and dynamic approaches to leverage their complementary advantages. Typically, constructs undergo initial static culture to facilitate cell attachment and matrix deposition, followed by transfer to perfusion systems to enhance nutrient delivery and oxygenation while minimizing early mechanical stress. Ahmed et al. developed a 3D human liver system by seeding cells onto hollow fiber membranes made of polyethersulfone. Cells within the membrane system were initially cultured under static conditions for 2 days and subsequently maintained under dynamic conditions using a bioreactor for 28 days [56].

Overall, maturation strategy selection depends on construct thickness, vascular architecture, cell source, and material properties. While static culture remains suitable for small-scale and early-stage studies, perfusion-based, hybrid, and large-scale bioreactor systems are increasingly essential for achieving physiologically relevant liver tissues. As liver tissue engineering advances, integration of fabrication and maturation strategies will be critical for translating engineered constructs toward clinically relevant applications.

#### 3.2.4. Volumetric Scalability

Achieving clinical efficacy in IALs requires a functional volume of approximately 200 mL, integrated with a hierarchical vascular network. However, as summarized in Table 7, most current prototypes remain limited to sub-milliliter scale. This discrepancy often arises from physical constraints inherent to their fabrication technique or their focus on initial proof-of-concept validation. To evaluate the feasibility of organ-scale replacement, it is crucial to distinguish between the current construct volume and the scalability potential of a given platform. In Table 7, authors have tried to assess various IAL platforms not only by their demonstrated dimensions but also by the specific scalability strategies employed to overcome existing volumetric barriers.

### 3.3. Functional Readiness for Implantation

The development of a fully functional bioartificial liver remains a long-term goal, and standardized criteria for evaluating the functional readiness of 3D liver constructs for liver implantation have yet to be established. 3D liver constructs can be evaluated through liver-specific functional assessments and in vivo studies in animal models before implantation in the human body. Although numerous biomarkers exist to evaluate liver condition and hepatocellular functions, these metrics alone do not reflect how well an artificial construct performs at the tissue or organ level scale. For this reason, this section assesses 3D liver constructs based on how closely they recapitulate the microenvironments of the native human liver. Here, microenvironmental features are treated not as design variables, but as functional benchmarks indicating whether engineered constructs have reached implantation-relevant maturity.

**Figure 3:**
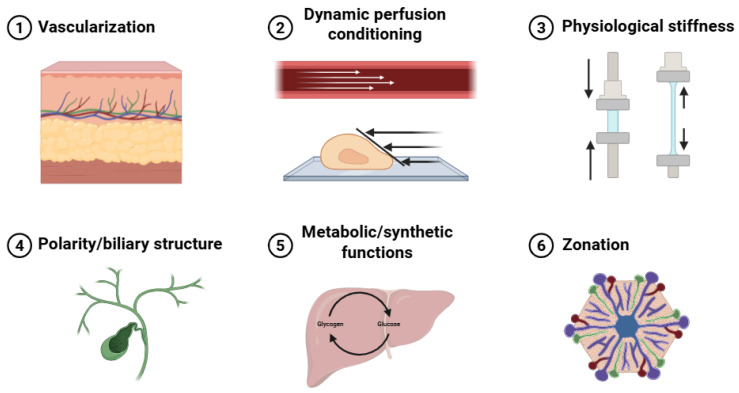
Liver microenvironments: vascularization, dynamic perfusion conditioning, physiological stiffness, polarity and biliary structure, metabolic and synthetic functions, and zonation.

Six essential hepatic physiological features were identified as key evaluation criteria: vascularization, dynamic perfusion conditioning, physiological stiffness, polarity and biliary structure, metabolic and synthetic functions, and zonation (Figure 3). Of the 71 initially screened articles, 54 provided relevant hepatic physiological data. The other 17 studies [24,34,36,40,43,46,49,59,61,65,71,72,75,80,81,83,86] could not be analyzed in this section as they prioritized platform development, hepatic differentiation, or basic biocompatibility over comprehensive functional evaluation. Table 8 summarizes the six key hepatic microenvironmental features, together with representative evaluation methods, functional implications, and limitations, used to assess in vitro hepatic functionality and implantation readiness of 3D bioartificial liver constructs.

#### 3.3.1. Vascularization

Vascularization in implantable liver constructs can be evaluated across hierarchical levels, ranging from endothelial cell presence and structural organization to functional perfusion, vascular maturation, and host–graft integration. Approximately 33% of the included studies focused primarily on vascular formation [21,27,32,33,39,41,44,48,53,55,56,68,74,76,77,78,89], reflecting its central role as a defining microenvironmental feature for implantable 3D liver constructs. Vascular networks directly regulate oxygen and nutrient transport, thereby determining diffusion limits, metabolic stability, and intratissue cell viability within 3D constructs.

All included studies validating vascular formation employed endothelial immunostaining, most commonly targeting CD31 (also known as PECAM-1) [21,27,32,33,39,41,44,48,53,55,56,68,74,76,77,78,89], to confirm endothelial identity and vessel-like structure formation. However, while endothelial identity was consistently confirmed, only a limited subset of studies assessed markers of endothelial maturation [41,53,74]—such as tight junction formation (e.g., ZO-1, claudin-5), basement membrane deposition (collagen IV, laminin), flow-responsive signaling (eNOS, KLF2), or perivascular cell recruitment—thereby limiting conclusions regarding vascular stability and implantation readiness. For spatial visualization, confocal fluorescence microscopy was the predominant modality used to analyze immunostained constructs in vitro [21,27,33,39,44,55,56,68,74,77,89], enabling 3D assessment of vessel continuity, branching, and lumen formation, but not long-term perfusion stability. In in vivo studies, histological staining, primarily hematoxylin and eosin (H & E), was additionally employed to evaluate vascular remodeling, host–graft integration, and perfused vessel morphology following implantation) [27,41,44,53,55,74,76,77,78,89].

Notably, 5 studies demonstrated that engineered vascular architectures enabled the formation of thicker viable tissue constructs by overcoming diffusion limitations [27,32,55,56,68], resulting in improved hepatocellular performance—including enhanced albumin secretion [27,32,55,56,68], urea synthesis [32,56], and CYP450 activity [27,68]—when compared with non-vascularized controls.

11 studies reported evidence of angiogenesis or host–graft vascular integration [21,32,33,39,53,68,74,77,78], assessed through indicators such as endothelial sprouting [39,77], inosculation with host vasculature [74,77], or sustained perfusion patency following implantation [39,53,74,77]. In contrast, 2 studies focused on pre-vascularization strategies, demonstrating the formation of vascular networks prior to implantation but did not include in vivo implantation or integration data [21,77].

Collectively, these findings indicate that although vascular formation is widely achieved, the limited assessment of endothelial maturation and functional integration remains a major barrier to predicting post-implantation vascular stability.

#### 3.3.2. Perfusion and Flow Conditioning

Perfusion-based culture strategies were evaluated across hierarchical levels, ranging from the implementation of dynamic flow to physiologically relevant shear exposure, flow-induced functional enhancement, and long-term stabilization of hepatic function in thick 3D constructs. This microenvironmental feature is particularly important to evaluate in long-term cultures and in thick 3D constructs, where diffusion alone is insufficient to sustain cell viability. Adequate delivery of oxygen and nutrients is essential as engineered liver tissues approach clinically relevant scales, and perfusion becomes a key determinant of functional stability.

Across the included literature, 12 studies implemented dynamic perfusion conditioning using a range of platforms, including microfluidic chips [23,29,51], perfusion bioreactors [22,52,73], tubular perfusion systems [45,64,82], or sinusoid-mimicking flow platforms [25,30,35,52]. Flow was generated primarily using peristaltic circulation pumps [22,25,35,45,52,64,73], linear syringe pumps [23,29,30], or unidentified sharkers [82].

Functional benefits of flow conditioning were evaluated in 7 studies over culture periods ranging from 7 to 30 days [22,23,25,29,30,46,64]. Reported improvements included increased albumin or urea secretion [22,23,25,29,30,64], enhanced glucose consumption [22,23], elevated CYP450 activity [29,64], or improved polarity markers [45].

Despite these functional gains, quantitative characterization of shear stress was reported in only a small subset of studies [30,52,64], likely reflecting the challenges associated with accurately calculating local shear forces within complex 3D geometries. Notably, Watanabe et al. directly investigated the influence of shear stress on microvessel formation using fluorescence-based imaging [52]. Instead, 4 studies selected flow rates based on estimated oxygen consumption requirements rather than explicitly defined mechanical parameters [23,25,45,82].

These studies demonstrate that dynamic perfusion primarily supports long-term viability and metabolic stability in engineered liver tissues, while precise control of physiologically relevant shear stress and integration with vascular maturation remain unresolved challenges for implantation-ready constructs.

#### 3.3.3. Physiological Stiffness

Under healthy conditions, native human liver tissue exhibits a low elastic modulus of approximately 0.4–2.0 kPa [57], whereas pathological conditions such as fibrosis are associated with markedly increased stiffness. Consistent with this, hepatocytes cultured on substrates outside the physiological stiffness window frequently exhibit dedifferentiation, fibrotic marker expression, and impaired metabolic function, underscoring the importance of mechanical microenvironment control in engineered constructs.

Across the reviewed literature, 7 studies explicitly engineered the mechanical properties of the culture microenvironment using a variety of material systems, including decellularized ECM (dECM)-based matrices [42,50], fibrous protein-based construct [79], PEG-based hydrogel [57], gelatin-based hydrogel [70], chitosan-based matrix [62], and nickel–titanium-based construct [60]. Mechanical properties were characterized using uniaxial tensile or compressive testing [42,60,70,79], rheometry [50,62], atomic force microscopy (AFM) [57], and dynamic thermomechanical analysis [50].

At the functional level, Lee et al. demonstrated that tuning the elastic modulus to values close to native liver stiffness significantly improved hepatocellular function and metabolic activity [57]. Lu et al. further showed that construct stiffness is not static but evolves over time during culture due to hydration and matrix remodeling [70], and that inactivation of YAP/TAZ signaling under physiologically soft conditions promotes hepatocyte function. These findings highlight stiffness as a dynamic regulator rather than a fixed design parameter. However, despite these mechanistic insights, most studies limited their evaluation to maintaining constructs within a presumed physiological stiffness range, without systematically interrogating how deviations in stiffness affect long-term hepatic function, mechanotransduction stability, or implantation readiness [42,50,60,62,79].

#### 3.3.4. Hepatocyte Polarity and Biliary Structure

Once hepatocytes reach a mature state, the establishment of apical–basal polarity is essential for coordinating intracellular metabolic processing and enabling directional bile secretion through canalicular and biliary networks. Unlike albumin or urea secretion, polarity reflects spatial organization and vectorial transport, which are indispensable for implantation because bile accumulation or misdirection can rapidly lead to cytotoxicity, inflammation, and graft failure in vivo.

8 studies evaluated hepatocyte polarity and/or bile duct formation using engineered architectures, including PDMS-based microchannels [54], bioprinted channels [87], decellularized liver vascular channels [26], and angiogenesis-driven biliary structures [58,63,67,88,90].

Hepatocyte polarity was primarily assessed by immunostaining for nuclear transcription factors associated with epithelial differentiation, including nuclear transcription factors associated with hepatocyte differentiation (HNF4α, HNF1β) [58,67,88,90], apical transporters (e.g., MRP2) [54,63,88], or tight junction markers such as ZO-1 [54,88]. These markers were used to confirm apical membrane formation and canalicular boundary definition within 3D constructs.

In parallel, Bile duct or biliary epithelial formation was evaluated using established biliary markers, including CFTR [58,67,90], DPPIV [26,54], or cytokeratins (CK7/CK19) [63,88]. Notably, Lewis et al. demonstrated that bile duct morphogenesis was strongly influenced by geometric parameters, showing that variations in channel width and branching angle significantly affected duct alignment and continuity [87].

Despite robust structural characterization, most studies focused primarily on marker expression and morphology, with relatively few evaluating whether polarity or biliary formation translated into global hepatic function, such as albumin secretion or CYP450 activity [54,58,67]. Moreover, longitudinal evaluation remains largely absent. Only a single study investigated temporal changes in polarity development over extended culture [54], leaving open questions regarding the stability of biliary structures, their adaptability after implantation, and their resistance to remodeling or collapse under physiological flow and mechanical stress.

#### 3.3.5. Metabolic and Synthetic Functions

While microenvironmental features such as vascularization, perfusion, stiffness, and polarity establish the conditions for hepatocyte activity, metabolic and synthetic output represent the ultimate functional readout of an implantable bioartificial liver. These functions integrate cellular maturity, multicellular interaction, and microenvironmental fidelity, and therefore serve as the closest surrogate for native liver performance at the tissue level.

Importantly, unlike individual microenvironmental markers, metabolic and synthetic outputs reflect system-level functionality, making them indispensable for assessing whether engineered constructs can provide meaningful hepatic support after implantation.

Only 4 studies evaluated metabolic and synthetic liver functions using global biochemical markers, including total bile acids (TBA) [69,85], fibronectin production [84], and ammonia metabolism [38]. These markers were used to assess whether engineered constructs could reproduce higher-order hepatic processes beyond albumin secretion or urea synthesis alone.

Among these, 3 studies demonstrated overall improvement in liver function using combined cellular biomarkers and survival outcomes in liver failure models, indicating functional relevance at the organismal level [47,69,85]. These studies provide critical evidence that enhanced metabolic and synthetic performance in engineered tissues can translate into therapeutic benefit at the organismal level, which is a key requirement for implantable systems.

Despite their importance, integrated metabolic and synthetic functions were evaluated in a surprisingly small fraction of studies, and often only as secondary endpoints. Most reports relied on single-pathway readouts, rather than coordinated metabolic profiling or longitudinal functional assessment.

#### 3.3.6. Liver Zonation

In native liver tissue, hepatocytes are spatially organized along the sinusoidal axis into zone 1 (periportal), zone 2 (midzonal), and zone 3 (pericentral) regions, each characterized by unique gene expression profiles and functional specializations [91]. This phenomenon, known as liver zonation, enables the liver to efficiently coordinate complex and sometimes opposing metabolic processes within a single organ.

For implantable bioartificial livers, zonation is not merely a refinement of function but a marker of advanced tissue maturity, reflecting the successful integration of oxygen gradients, perfusion, and metabolic specialization. Constructs lacking zonation may exhibit baseline hepatic function, but they fail to recapitulate the spatial heterogeneity required for physiological relevance and long-term functional stability.

A total of 5 studies successfully induced zonation-like behavior in engineered liver constructs by establishing controlled oxygen or nutrient gradients, primarily using microfluidic platforms [37,91] or spheroid-based systems [28,31,66]. These approaches aimed to recapitulate the spatial heterogeneity of hepatocyte function observed along the native liver lobule.

Zonation was validated through differential CYP450 expression, visualized using heatmap analyses [28,31,91]. In parallel, oxygen consumption rates (OCR) supported by computational simulations were used to correlate metabolic activity with local oxygen availability and gradient distribution [37,66,91].

Beyond descriptive patterning, 2 studies demonstrated functional relevance, showing that zonation-mimicking constructs exhibited improved or differential drug responses, suggesting enhanced physiological fidelity for pharmacological evaluation [28,91]. Notably, Fortin et al. reported that zonation-like metabolic patterns were maintained over long-term culture, persisting for up to 9.5 weeks following implantation, highlighting the potential stability of engineered zonation in implantable liver constructs [31].

Despite its physiological importance, zonation remains one of the least explored and least standardized features in implantable liver engineering. Most studies relied on indirect metabolic markers and computational inference, with limited validation of multi-pathway coordination or interaction with other microenvironmental cues such as stiffness or biliary polarity. Moreover, only a single study assessed long-term persistence of zonation following implantation, highlighting a substantial gap between proof-of-concept demonstrations and translational relevance.

### 3.4. In Vivo Implantation Studies

In vivo studies are crucial for translating liver tissue engineering strategies into clinically viable therapies. Implantable liver constructs must demonstrate not only the retention of hepatic functions but also integration with host tissues, including vascularization and immunocompatibility, in animal models. Experiments in rodents and other animals allow assessment of survival benefit, graft functionality (e.g., albumin production, CYP450 activity), and systemic effects such as normalization of liver enzymes. Successful in vivo implantation outcomes provide proof-of-concept that an engineered liver construct can restore or support liver function in a living organism, a necessary step before clinical trials. Recent studies across various engineering approaches, from decellularized organ constructs to cell-laden hydrogels and bioprinted tissues, have shown promising results in animal models of liver injury or failure, underscoring the importance of in vivo evaluation for translation [34,50].

Among the 36 studies identified for in vivo evaluation of bioartificial liver systems, we categorized them into four groups based on biomaterials used, biofabrication techniques, cell type, and vascularized constructs. This classification allows for structured comparison of implantation strategies and biological performance across engineered liver systems. Table 9 summarizes key features and outcomes from each group.

#### 3.4.1. Hydrogel-Based Constructs (Non-Bioprinted)

Hydrogel-based liver constructs involve embedding liver cells in biocompatible hydrogels or capsules, creating an implantable cell delivery vehicle. These approaches use natural or synthetic hydrogels (e.g., collagen, Matrigel, alginate, chitosan, polyethylene glycol) to encapsulate hepatocytes or hepatic spheroids, often along with supportive cells such as MSCs to enhance engraftment. Hydrogels can be injected or implanted with minimal invasiveness, conforming to tissue spaces. A key design consideration is diffusion: hydrogel implants are typically kept small (or highly porous) to allow oxygen and nutrient transport until host vasculature grows in. Animal models have tested hydrogel constructs in both ectopic sites (such as subcutaneous or intraperitoneal locations) and orthotopically within injured livers.

Several studies illustrate the potential of hydrogel-based liver implants. Volvox spheres, for example, are an innovative dual-layer hydrogel microenvironment in which an outer sphere encloses smaller inner hydrogel spheres containing cells. Chang et al. [75] encapsulated both MSCs and murine hepatocytes (AML12 cells) in volvox spheres and implanted them into rats with retrorsine/CCl_4_-induced liver injury. The co-encapsulated MSCs differentiated into HLCs and secreted supportive factors, yielding improved liver function in vivo. Notably, rats receiving the cell-laden spheres showed significant drops in liver enzymes (ALT/AST) and evidence of new liver tissue formation and repair at the injury sites, compared to controls [75]. This demonstrates that hydrogel vehicles can deliver cells that actively contribute to regenerating damaged liver tissue. Similarly, injectable self-healing hydrogels have been explored for treating liver fibrosis. Tai et al. [62] developed a chitosan-phenol-based hydrogel that rapidly gels in situ and supports hepatocyte spheroids. In a rat fibrosis model, injection of this hydrogel into the liver resulted in alleviation of injury: within 2 weeks, serum AST/ALT ratios declined by ~28% and the fibrotic scar area was reduced by 70%, indicating regression of fibrosis and partial recovery of liver function [62]. The hydrogel provided a favorable microenvironment for the hepatocytes, promoting matrix remodeling and an anti-inflammatory response in the liver.

Another approach is to introduce engineered hepatic microtissues into the liver via hydrogels. Deng et al. reported that multiple injections of collagen-based micro-constructs containing neonatal liver cells into cirrhotic rat livers led to improved liver architecture and function [44]. Co-cultures of hepatocytes with stromal cells in hydrogels have also shown enhanced engraftment: encapsulating cells in a soft matrix can prevent cell washout and provide initial trophic support until host vessels integrate. Overall, non-bioprinted hydrogel constructs have achieved weeks to months of in vivo function, with outcomes such as sustained albumin secretion, normalization of liver enzymes, and even fibrosis reversal [48]. These studies underscore the versatility of hydrogels as cell carriers that can be tailored for specific liver repair applications (injectable therapies, tissue patches, etc.) and emphasize the need for strategies to promote rapid vascularization of the implants.

Decellularized liver constructs leverage the native liver ECM and vascular architecture to support implanted cells. Typically, whole livers from animals (rodents or pigs) are decellularized to remove cellular components while preserving the ECM framework and perfusable vascular channels. These acellular liver matrices are then repopulated with liver cells, often primary hepatocytes or hepatocyte progenitors, and sometimes endothelial cells to line the vasculature, before implantation. Rodent models have been used to test the implantation of recellularized liver constructs orthotopically or as auxiliary grafts. For example, early proof-of-concept studies demonstrated that a decellularized rat liver construct seeded with hepatocytes could be transplanted and maintain brief hepatic function in vivo, though limited by thrombosis and poor long-term graft survival due to incomplete endothelialization [38,89]. Subsequent advances addressed these issues by pre-endothelializing the vasculature: re-endothelialized decellularized liver constructs showed improved perfusion and cell engraftment after transplantation in animal models [38]. Decellularized liver constructs provide liver-specific biochemical cues and an intact 3D lobular structure, thereby helping maintain hepatocyte function in vivo. However, ensuring immediate and stable graft perfusion remains a challenge, as xenogeneic matrices can trigger thrombosis or immune reactions if not fully endothelialized and if residual DNA remains [48].

In addition to whole-organ grafts, liver dECM has been fashioned into 3D constructs for localized repair. A recent study used decellularized liver matrix as a component of a 3D-bioprinted construct, combining it with gelatin and sodium alginate to fabricate a transplantable liver construct. This 3D-bioprinted liver, loaded with primary hepatocytes, was heterotopically implanted into the mesentery of mice subjected to either 90% partial hepatectomy or a fumarylacetoacetate hydrolase (Fah)-deficiency model of liver failure. The construct supported hepatic function, enabled rapid vascular integration via pre-embedded artificial vessels, and significantly prolonged survival compared to untreated controls [50]. These findings underscore the utility of ECM-derived bioinks not only in structural replication but also in facilitating functional integration in severe liver failure models.

#### 3.4.2. 3D-Bioprinted Liver Constructs

3D bioprinting enables the fabrication of liver constructs with precise architecture, including the placement of multiple cell types in defined patterns and the incorporation of perfusable channels. Bioprinted liver tissues typically use bioinks composed of hydrogels such as gelatin-methacrylate, collagen, or alginate) mixed with hepatic cells, and sometimes endothelial or stromal cells. The printed constructs, often on scales ranging from a few millimeters to centimeters, aim to mimic features of liver microarchitecture (such as lobule-like arrangements or sinusoidal networks) to enhance functionality. In vivo implantation of bioprinted tissues is a recent but rapidly advancing area, with studies demonstrating that printed liver constructs can survive, integrate, and partially assume liver functions in animal models.

A notable example is the 3D-bioprinted hepatic tissue reported by Lee et al. [79], who used a collagen-based bioink to create “hepatic blocks” composed entirely of human ADSCs and their differentiated hepatocyte-like progeny. These printed liver blocks, stabilized with a natural crosslinker, genipin, were transplanted into the livers of immunodeficient rats with acute liver failure. Over 4 weeks, the implants remained intact and cells migrated into the host parenchyma. Rats receiving the bioprinted tissue showed recovery of liver function, including normalization of serum biochemistry (e.g., liver enzyme levels returning to near normal) [79]. Human-specific albumin was detected in the rat circulation, confirming that the human stem cell–derived hepatocytes in the printed construct survived and functioned in vivo. Notably, inflammatory cytokine profiling indicated an increase in IL-10 in treated rats, suggesting the printed stem-cell constructs also conferred immunomodulatory benefits [79]. This study demonstrated both the safety and efficacy of a bioprinted stem-cell hepatic graft in rescuing acute liver failure.

More recently, Deng et al. described a 3D-bioprinted liver construct with a built-in vascular channel that markedly improved survival in mouse models of liver failure [50]. In this study, primary hepatocytes were printed in a tailored bioink alongside an artificial blood vessel structure. When transplanted heterotopically into the mesentery of mice with fatal liver injuries, the bioprinted liver construct rapidly grafted to the host. The presence of the pre-designed vessel facilitated anastomosis with the host circulation, leading to timely oxygenation and nutrient supply to the implant [50]. Treated mice showed extension of survival and recovery of liver function (e.g., improved metabolic detoxification and synthesis), whereas untreated controls succumbed to liver failure [50]. This is evidence that bioprinted liver tissues can functionally integrate into a living system when provided with a sufficient vascular network.

#### 3.4.3. Stem-Cell–Derived Hepatic Constructs

Stem-cell–derived hepatic constructs encompass engineered tissues where the functional cells are derived from stem cells or progenitors, including pluripotent stem cells (ESCs/iPSCs), adult stem/progenitor cells, or fetal liver cells. The use of stem cell–derived hepatocytes (or hepatoblast-like cells) is attractive for generating clinically scalable grafts, since primary hepatocytes are often scarce and do not proliferate. In these constructs, stem cells are typically differentiated in vivo into hepatocyte-lineage cells or organoids, which are then implanted, either alone or within a construct, to test their maturation and function inside a living organism. A key consideration is that stem cell derivatives should further mature in vivo and not form teratomas. Animal implantation studies have investigated the engraftment and function of such constructs, often in models of liver injury that create a permissive niche for new hepatocytes.

Recent advances in liver tissue engineering have leveraged the regenerative potential of stem cells, particularly ADSCs, in combination with innovative biomaterials. A striking example is the use of plant-derived cellulose constructs derived from decellularized apple tissue, which possess a lobule-like porous structure favorable for hepatic regeneration. Hu et al. [80] demonstrated that ADSCs seeded onto these constructs differentiated into HLCs in vitro and, upon implantation into mice with acute liver injury, promoted liver function recovery and vascular and bile duct neogenesis. Similarly, Wang et al. [72] introduced an innovative liver tissue engineering strategy by recellularizing decellularized celery-derived constructs withhiPSCs–derived hepatocytes (hiPSC-Heps). Inspired by the natural lobule-like microarchitecture of celery stems, the construct provided interconnected porous channels suitable for hepatic cell engraftment and proliferation. In vitro, the construct supported 3D spheroid formation and preserved hepatic function, as evidenced by elevated albumin secretion, glycogen storage, and expression of hepatic genes and proteins. When transplanted into the liver, spleen, or omentum of nude mice, the constructs demonstrated excellent biocompatibility, formation of vascular-like structures, and maintenance of hepatic function, with ALB- and PAS-positive cells detected within the grafts. These results underscore the potential of plant-derived constructs as a cost-effective and bioactive platform for stem cell–based liver regeneration.

Deng et al. developed a pre-vascularized liver tissue (PLT) from human pluripotent stem cells by first differentiating the cells into hepatic progenitors within tiny hydrogel microspheres, then assembling these microspheres with endothelial cells to form a 3D tissue [44]. When implanted intrahepatically into NOD/SCID mice, the stem cell–derived liver tissue exhibited robust engraftment: the presence of pre-formed microvessels promoted rapid integration with the mouse vasculature, resulting in significantly higher human albumin levels in the blood compared to non-vascularized cell grafts [44]. Histology confirmed that the engineered tissue had inosculated with host vessels and the stem cell–derived hepatocytes further matured in vivo, contributing to liver function.

Collectively, these studies underscore the potential of stem-cell–derived hepatic constructs, especially when integrated with bioactive or biomimetic constructs, to restore liver function and reconstitute complex hepatic architecture in vivo.

#### 3.4.4. Perfusable/Vascularized Bioengineered Devices

Because hepatocytes are highly metabolic and oxygen-demanding, engineered liver constructs benefit greatly from rapid vascularization or even pre-existing perfusion channels. Perfusable or vascularized bioengineered devices are implantable constructs that incorporate design features to facilitate blood flow and oxygen delivery to cells. These can range from macro-scale encapsulation devices with built-in vasculature, to porous constructs seeded with supportive cells to induce angiogenesis, to perfusion chambers connected to host circulation. The goal is to create an implant that can be surgically placed in the body and quickly establish blood vessel connections, thus supporting a larger mass of liver tissue with sustained function.

One illustrative design is the dual-compartment hydrogel device described by Seale et al. [48] for minimally invasive hepatocyte transplantation. This implant consists of a hollow core loaded with primary hepatocytes, surrounded by an outer macroporous hydrogel, composed of PEGDA and hyaluronic acid, that can host supportive cells and invite host vessel ingrowth. When implanted subcutaneously in NOD/SCID mice, the dual-compartment device became well-vascularized in the outer compartment and successfully sustained the human hepatocytes in the core for at least one month [48]. Evidence of function was confirmed by the presence of human albumin in the mice’s circulation and immunostaining of the retrieved device showing albumin-producing hepatocytes that remained viable in vivo [48]. The pre-designed porosity of the device enabled anastomosis of host blood vessels, thereby creating an ectopic perfused liver module under the skin. Such an approach highlights how biomaterial engineering can overcome diffusion limitations and support long-term engraftment of liver cells outside the native liver.

Inducing vascularization in bioengineered liver grafts is challenging, as shown by some less successful attempts. Lee et al. implanted collagen-based constructs seeded with human ADSCs onto the serosal surface of the stomach in mice, to develop an angiogenic bioartificial liver tissue in situ [78]. While the constructs adhered to visceral surfaces, after 30 days, the investigators found no evidence of blood vessel formation within the constructs and no detectable expression of angiogenic markers (VEGF, CD34, CD105) [78]. The implanted stem cells survived within the collagen matrix and the material was biocompatible, but the absence of intrinsic vascularization limited further development of hepatic tissue. This outcome underlines the need for either pre-vascularizing constructs or providing strong angiogenic stimuli when designing implantable liver devices. Strategies being explored to enhance vascularization include incorporating endothelial cells or pro-angiogenic factors into the construct, using oxygen-generating biomaterials, or connecting constructs directly to arterial flow.

In summary, perfusable and vascularized liver devices show great promise as auxiliary liver support systems. By ensuring blood supply either via material design or co-culture with vasculature-forming cells, these implants can be scaled up in thickness and cell density, potentially achieving clinically relevant graft masses. Ongoing in vivo work suggests that a combination of design elements, such as porous matrices, pre-seeded endothelial networks, and strategic placement in well-vascularized host sites, is critical to achieve long-term survival and function of implanted liver tissues.

## 4. Conclusions

The research paradigm for artificial livers is rapidly transitioning from temporary extracorporeal support to biofabricated, vascularized constructs designed for permanent functional integration. While significant progress has been made in hiPSC sourcing and bioactive construct design, scaling these prototypes to clinically relevant volumes remains a formidable challenge. Specifically, the technological gap between current 1 cm^3^ models and the required 200 mL threshold underscores the urgent need for enhanced volumetric scalability. Success further depends on integrating hierarchical vascular networks exceeding 2 mm in diameter for surgical anastomosis alongside functional biliary trees for long-term graft stability. Future directions should leverage high-resolution multicellular patterning and microfluidic induction to refine hepatic microarchitecture and spatial zonation. Furthermore, advancements in autologous stem-cell reprogramming offer promising pathways to minimize immunogenicity and improve clinical eligibility. Ultimately, establishing standardized benchmarks for functional readiness will be vital for translating these bioengineered constructs into transformative clinical therapies.

## Figures and Tables

**Table 1:** The specific search queries utilized in the three databases.

Database	Search Queries
Web of Science	TS = (“artificial liver” OR “liver tissue engineering” OR “engineered liver”) AND LA = (English) AND PY = (2015–2025) AND DT = (Article) NOT DT = (Review OR “Meeting Abstract” OR “Editorial Material” OR “Conference Paper” OR “Proceedings Paper” OR “Early Access” OR “Retracted Publication” OR “Book Chapter”)
PubMed	(“artificial liver” [tw] OR “liver tissue engineering” [tw] OR “engineered liver tissue” [tw]) AND (“2015/01/01” [dp]: “3000” [dp]) AND (English [lang]) AND (journal article [pt]) NOT (review [pt] OR systematic review [pt] OR meta-analysis [pt]) NOT (editorial [pt] OR comment [pt] OR published erratum [pt] OR retracted publication [pt] OR retraction of publication [pt])
Scopus	TITLE-ABS-KEY(“artificial liver” OR “liver tissue engineering” OR “engineered liver”) AND PUBYEAR > 2014 AND LANGUAGE(“English”) AND DOCTYPE(ar) AND NOT DOCTYPE(re OR cp OR ch OR ed OR no OR er OR rp)

**Table 2:** Summary of species-derived cell types used.

Species	Cell Type/Cell Line	Cell Density	References
Human	HepG2/Hepatoblastoma cells	1.5 × 10^5^–4.5 × 10^7^ cells	[14,21,22,23,24,27,28,29,30,36,37,38,39,40,41,42]
Human	Human umbilical vein endothelial cells (HUVECs)	1 × 10^4^–5 × 10^7^ cells	[21,23,24,25,26,27,28,29,30,31,32,33,36,39,40,43,44,45,46,47,48,49,50,51]
	Green fluorescent protein labeled-HUVECs (GFP-HUVEC)	2 × 10^6^ cells	[52]
	RFP-HUVECs	1 × 10^6^ cells	[49]
Human	EA. hy926 endothelial cells	1 × 10^6^–35 × 10^7^ cells	[14,41,53,54]
Human	Primary hepatocyte cell	1.04 × 10^4^–20 × 10^7^ cells	[25,27,31,38,43,45,48,55,56,57,58]
Rat	Primary hepatocyte cell	3.8 × 10^4^–8 × 10^6^ cells	[26,32,33,34,58,59,60,61,62,63,64,65,66]
Mouse	Primary hepatocyte cell	1.6 × 10^5^–8 × 10^7^ cells	[42,49,50,67,68,69,70]
Human	Normal human dermal fibroblast cell/NHDF	2 × 10^5^–17 × 10^6^ cells	[21,27,31]
	Normal human diploid fibroblast TIG-118 cell line	2.29 × 10^4^ cells	[55]
Human	Human iPSCs (hiPSCs)	5 × 10^4^–1.2 ×10^7^ cells	[44,51,71,72,73]
Rat	Bone marrow MSCs (BM-MSCs)	0.9 × 10^5^–1.95 × 10^7^ cells	[64,66,74,75]
Human	BM-MSCs	10^4^–0.3 × 10^6^ cells	[43,45,47]
Mouse	C166 (mouse endothelial cell)	5 × 10^6^ cells	[68]
Rat	WB-F344 hepatic oval (ratOv) cell	1 × 10^5^ cells	[35]
Mouse	Swiss 3T3 cells	0.8 × 10^6^ cells	[23,59]
	3T3-J2 fibroblast cell	0.48 × 10^5^ cells	[63]
Rat	H4-II-E-C3 rat hepatoma cells	1 × 10^6^ cells	[64]
Rat	Fetal liver cell	4 × 10^3^ cells	[34]
Human	Stellate cells	4 × 10^3^–10^6^ cells	[36,51,56]
Human	Human lung fibroblasts	7.5 × 10^4^ cells	[32]
Rat	Novel off-liver progenitors of liver sinusoidal endothelial cells (LSECs)	1 × 10^6^ cells	[35]
	Rat LSECs	2 × 10^4^–5 × 10^6^ cells	[26,64,76]
Human	HLCs differentiated from iPSCs and human embryonic stem cells (hESCs)	5 × 10^7^ cells	[44]
	Embryonic stem cell-derived hepatocytes (ESC-Hep)	1 × 10^7^ cells	[57]
Human	Liver Sinusoidal Endothelial Cells LEC (TMNK-1) cells	5 × 10^3^ cells	[77]
	Immortalized human hepatic sinusoidal EC-SV40	1.42–6 × 10^6^ cells	[22,59]
		5 × 10^6^ cells	[57]
	Novel off-liver progenitors of liver sinusoidal endothelial cells (LSECs)	10^6^ cells	[35]
Human	Adipose-derived stem cells (ADSCs)	1.7 × 10^4^–5 × 10^6^ cells	[29,78,79]
Rat	ADSCs	10^6^ cells	[80]
Human	L02 cells (HL-7702, human hepatocytes)	2 × 10^6^ cells	[81]
Human	AHLCs-hASC-induced HLCs	5 × 10^6^ cells	[79]
Mouse	Embryonic fibroblasts	1.06 × 10^6^ cells	[48,69]
Human	C3A cells	3 × 10^7^ cells, 2 mL pellet/9 mL medium	[54,82]
Rat	Biliary epithelial cells (BECs)	2.5 × 10^6^ cells	[58]
Mouse	Bipotential mouse embryonic liver (BMEL) cells	10 × 10^6^ cells	[83]
Human	Hepatocarcinoma (Huh 7.5) cell line	4 × 10^7^ cells	[84]
Rat	Hepatic stem cells	2 × 10^4^ cells	[65]
Human	Human bipotent HepaRG cells	Not specified	[85]
	HepaRG cells	2 × 10^5^–1 × 10^7^ cells	[57]
Mouse	NG2 + HPCs	3 × 10^7^ cells	[86]
Mouse	Immortalized mouse small cholangiocytes	Not specified	[87]
	SV40SM	5 × 10^4^–1.5 × 10^6^ cells	[88]
Mouse	Murine endothelial cells	2.5 × 10^8^ cells	[89]
Human	HEK293T viral producer cells	1.8 × 10^7^ cells	[25]
Human	Human umbilical cord blood-derived MSCs	2.9 × 10^6^ cells	[30]
Human	Human hepatocyte-derived liver progenitor-like cell	9.43 × 10^6^ cells	[46]
Mouse	Liver ductal organoids	5 × 10^6^ cells	[67]

**Table 3:** Summary of cell type combinations used in studies.

Number of Cell Types	Cell Type Combination	References
4	Hepatocytes + endothelial cells + myoblasts + viral producer cells	[25]
	hiPSCs + endothelial cells + stellate cells + macrophages	[51]
	Hepatocytes + MSCs + endothelial cells + fibroblasts	[48]
3	Hepatocytes + endothelial cells + stellate cells	[36,56]
	Hepatocytes + endothelial cells + fibroblasts	[21,23,27,31,32,59]
	Liver sinusoidal progenitor cells + hepatic oval cells	[35]
	Liver progenitor cells + hepatocytes + endothelial cells	[57]
	Hepatocytes + endothelial cells + vascular pericytes	[33]
	Hepatocytes + endothelial cells + stem cells	[29,30,43,45,64]
	Hepatocytes + cholangiocytes + fibroblasts	[63]
2	Cholangiocytes + endothelial cells	[87]
	Polydendrocytes (NG2) + hematopoietic progenitor cells (HPCs)	[86]
	Hepatocytes + fibroblasts	[55]
	Hepatocytes + endothelial cells	[14,22,24,26,28,39,40,41,44,49,50,53,54,68]
	MSCs + endothelial cells	[47]
	Hepatocytes + liver ductal organoids	[67]
	Fetal liver cells + hepatocytes	[34]
	Hepatocytes + biliary epithelial cells	[58]
	Hepatocytes + MSCs	[65,66,75,79]
	Liver progenitor cells + hepatocytes	[82]
1	Endothelial cells only	[52,76,77,89]
	hiPSCs only	[71,72,73,90]
	MSCs only	[74,78,80]
	Hepatocytes only	[37,38,42,60,61,62,69,70,81,84]
	Cholangiocytes only	[88]
	Liver progenitor cells only	[85]
	Embryonic liver cells only	[83]

**Table 4:** Comparison of the various material sources applied in artificial liver tissue engineering.

Materials		Crosslinking/Stabilization	Printability	Proliferation Capacity	Limitation	References
Nature hydrogel	Alginate	Ionic (Ca^2+^) or covalent (Ba^2+^, etc.)	Excellent	Low cell-adhesion	Relatively slow degradation requires modification for cell-adhesion	[57,64]
	Gelatin/ GelMA	Thermal (gelatin) + photo (GelMA, methacrylate)	Good	Good cell attachment and spreading	Low Stability: Melts/degrades rapidly at 37 °C, poor mechanicals	[27,68,70]
	Collagen	Thermal/self-assembly	Challenging	Excellent cell migration	Weak mechanics, slow gelation, needs reinforcement or crosslinking	[31,37,45,78]
	Fibrin	Enzymatic (thrombin + fibrinogen)	Good for cell encapsulation	Excellent cell migration, supports angiogenesis	Biodegrades rapidly, requiring stabilizing agents	[31,69]
Synthetic polymers	PEG: poly (ethylene glycol)/ PEGDA: PEG-diacrylate	Photo-cross-linkable, chemistry	Excellent	Poor cell-adhesion (surface treatments needed)	Lacks bioactivity, penetration of the oxygen and nutrient	[33,64,83,84]
	PLLA: poly-L-lactic acid/PCL: Poly-carpo-lactone	Radiation crosslinking/thermal chemistry	Excellent	Poor cell-adhesion (surface treatments needed)	Slow degradation, low bioactivity and hydrophobicity	[22,23,29]
	PES (polyether sulfone)	Photo-cross linkable/radiation crosslinking	Excellent	Poor cell-adhesion (surface treatments needed)	Lacks bioactivity, non-biodegradable nature	[56]
dECM	Porcine dECM	Thermal/self-assembly	Challenging, often low viscosity, needs blends	Contains numerous cell-binding domains, highly promotes cell attachment, differentiation, and long-term function	Weak mechanical strength, needs reinforcement, immunogenicity	[14,74,87,88,89]
	Mouse/Rat DECM					[26,35,41,52,71,86]
	Matrigel			Excellent cell migration, contains the key components of the basement membrane and growth factors	Poor structural and mechanical stability	[58,90]

**Table 5:** Construct structure and properties.

Structure/ Technique	Ref.	Vascularization		Presence of Bile Duct	Outcomes	Implantation/ Location
		Mirco-Vascular Network	Over 1 mm Vessel			
Layered/ cell-sheet	[55]	Yes	No	Yes	Produce vascularized subcutaneous human liver tissue (VSLT) in vivo using engineered hepatocyte/fibroblast sheets (EHFSs) without stem cells or ECs Maintain liver-specific functions and reconstructed structures	Mouse– Subcutaneous
	[27]	Yes	No	Yes	Create a vascularized liver tissue model using limited CPHs and co-culture of HUVECs/NHDFs via the layer-by-layer technique Evaluate the vascularized tissue after engrafting in mice	Mouse– Subcutaneous
	[51]	Yes	No	Yes	3D-imprinted cell-sheet technology to build biomimetic hepatic lobules Vascular endothelial cells infiltrate the hollow regions 3D-imprinted vascularized lobules support drug screening and clinical liver-tissue engineering potential	Rat–Liver
Spheroid/ organoid/ self assembling	[69]	Yes	No	No	Rapid self-assembly mini liver (RSAL) formed using natural coagulation of fibrinogen and thrombin RSALs develop a functional vascular system connected to the host mouse within 14 days after mesentery implantation	Mouse– Mesentery
	[65]	No	_	No	Nanostructured self-assembling peptide–coated bioreactor supports liver stem-cell expansion and hepatic differentiation Performance is superior to Matrigel	*No comment about implantation*
	[63]	No	_	Yes	Hepatic microtissue is formed by coculturing rat primary hepatocytes with cholangiocytes and stromal cells Hepatocytes in spheroids-maintained viability and function for up to 7 days Hepatocytes became polarized, forming bile canaliculi with tight junctions	*No comment about implantation*
	[31]	Yes	No	No	Built engineered human liver tissues and analyzed engraftment, expansion, and metabolic phenotype over time after implantation Grafted tissues showed progressive, spatially restricted expression of key functional proteins matching native liver zonation First demonstration of engineered human liver tissue zonation in vivo, with important translational relevance	Mouse–Liver
Sacrificial templating/embedded molds	[53]	Yes	Yes	No	Re-endothelialization of porcine liver constructs achieved by heparin–gelatin vessel precoating Blood vessel surface modification enhanced EC attachment, migration, and anti-platelet properties HepG2 cells showed higher function in HG-precoated constructs both in vitro and after in vivo transplantation	Hybrid pigs–Liver
	[74]	No	No	No	Hepatic grafts survived, maintained hepatocyte-specific functions, and anastomosed with host vasculature Cells self-organized into cord-like structures in vivo First report of long-term vascularized hepatic parenchyma at ectopic sites using decellularized liver constructs and stem cells	Rat–peritoneal cavity
	[87]	No	No	Yes	Complex biliary tree formation controlled in vitro using 3D-printed dECM hydrogels and sacrificial supports System enables 3D patterned co-culture of hepatocytes and cholangiocytes Cell lines demonstrate feasibility of directing duct formation while maintaining patterned hepatocyte co-culture	*No comment about implantation*
	[41]	Yes	No	No	Conjugated homogenized liver ECM into a decellularized rat liver to enhance structural and functional properties Conjugated constructs showed improved cellular spreading and viability compared to non-conjugated constructs Intrahepatic transplantation resulted in higher recruitment of hepatic regenerative cells and improved angiogenesis	Rat– Subcutaneous, Liver
Constructs (whole-organ or partial)	[21]	Yes	Yes	No	Successful fabrication of a complex in vitro triple-culture hepatic sinusoid model Cell–cell interactions in the model enhanced albumin secretion Core–shell bioprinting shown to be a useful tool for studying interactions and creating tissue-like models	*No comment about implantation*
	[46]	Yes	No	Yes	Silk fibroin–collagen I (SFC) were used to engineer hepatic lobule–like constructs with iHepLPCs and endothelial cells in dynamic culture iHepLPCs formed hepatic plate–like structures and differentiated into mature hepatocytes with improved functions in vitro and in vivo Co-culture generated hepatocyte–endothelial interfaces with better polarity, bile canaliculi formation, and enhanced liver functions compared to monocultures	Mouse– Subcutaneous
	[79]	No	No	No	Differentiated human adipose stem cells (hASCs) into HLCs and fabricated liver-regenerative hepatic block constructs via 3D cell printing with type I atelo-collagen hASCs and AHLCs migrated into the portal vein regions of hepatic lobules in rats within 4 weeks Serum biochemistry normalized in acute liver failure rats after construct transplantation	Rat–Liver
	[50]	Yes	Yes	No	Constructed 3D-bioprinted liver using in vitro–expanded primary hepatocytes with strong safety and functional robustness Developed bioinks with optimized mechanical, rheological, and printing properties for 3D bioprinting 3D-bioprinted liver restored lost liver functions and extended survival in liver-injured mice	Mouse–Liver
Microfluidic “liver-on-chip”/ perfused bioreactors	[73]	No	No	No	Built an artificial liver prototype (SHiNTA) using a perfusion bioreactor and liver microstructure made from hiPSC-derived hepatocytes based on a modified Blackford protocol SHiNTA artificial liver showed positive signs of cell maturation within the construct	*No comment about implantation*
	[37]	No	No	No	Designed a sinusoid blood flow–mimicking (SBF) liver microsystem to improve nutrient transport for large-scale, high-density 3D hepatocyte culture Assessed system performance via metabolite production, protein synthesis, and bilirubin detoxification using collagen and alginate ECMs Observed liver-rope–like structures and sphere-like hepatocyte clusters	*No comment about implantation*
	[43]	Yes	No	No	Biomimetic hepatic lobules with coaxial through-pores were engineered using microfluidic technology with parallel capillary installation Spatially anisotropic cell arrangement closely mimicked native hepatic architecture In situ transplantation in rat liver enhanced regeneration and reduced necrosis	Rat–Liver
	[44]	Yes	No	Yes	Generated COL1 hydrogel microspheres encapsulating hESCs via microfluidics and differentiated them into HLC microspheres HLC microspheres self-assembled with endothelial cells to form dense PLT resembling native liver tissue PLT successfully engrafted into mouse liver and showed improved hepatic function in vivo	Mouse–Liver
	[54]	Yes	No	Yes	Used microfluidic chip technology with natural alginate hydrogels to create 3D liver tissues mimicking hepatic plates Increased culture duration enhanced viability, function, polarity, mRNA expression, and ultrastructure 3D hepatic plate models promoted bile-secretion pathway changes via nuclear receptors and bile transport mechanisms	*No comment about implantation*

**Table 6:** Construct maturation strategies for artificial liver tissues.

Maturation Technique	Key Principle	Common Applications	References
Static culture system	Simple immersion in the medium	Standard baseline, low-cost, high-throughput screening	[14,21,27,31,32,33,34,36,38,40,42,44,47,48,49,50,51,54,55,57,58,59,60,61,62,65,66,68,69,70,72,74,77,79,80,81,83,84,85,88]
Dynamic culture with perfusion chamber/system	Continuous medium flow	Mimics vascular flow; enhances hepatocyte function and viability for larger constructs.	[22,23,26,29,30,35,41,45,52,53,64,71,73,89]
Dynamic culture with microfluidic device	Precise control of microscale flow and tissue architecture	Models’ sinusoid structure, cell–cell interactions, and high-fidelity mechano-stimulation.	[37]
Rotary culture system	Provides gentle mixing and low-shear suspension via rotation.	Promotes 3D aggregate/spheroid formation and uniform culture conditions	[24,46,86]
Dynamic culture with BAL	Extracorporeal perfusion system for clinical or large-scale assistance devices	Focus on scaling up for therapeutic translation and acute liver failure support.	[25,82]
Static and dynamic culture combined	Use both methods sequentially or parallelly	First establish tissue in static, then perfuse in dynamic.	[56]

**Table 7:** Volumetric scalability of liver construct fabrication techniques.

Fabrication Technique	Construct Volume	Scalability Potential	Scalability Strategies	References
Decellularization & Recellularization	<1 mL	Moderate to High	Perfusion-based systems for whole-organ scaling Automated bioreactor seeding protocols Modular vascular network recellularization Process standardization for production	[40,74,80]
	1–10 mL			[25,26,28,35,36,41,52,67,71,73,76,86]
	>10 mL			[22,53,89]
Extrusion-based 3D Bioprinting	<1 mL	Moderate	Multi-nozzle parallel printing systems Hybrid bioink development for faster printing Perfusable channel design and integration Layer-by-layer deposition scaling Automated print path optimization	[14,21,29,32,79,81]
	1–10 mL			[49,50]
DLP-based 3D Bioprinting	<1 mL	Moderate to High	Large-area projection systems Batch production of identical units Optimized photopolymer chemistry Modular design strategies	[64,70]
Self-Assembly/ Aggregation	<1 mL	Moderate to High	High-throughput spheroid/organoid production Suspension culture scale-up systems Modular tissue unit assembly approaches Controlled aggregation parameter optimization Scalable bioreactor integration	[23,33,34,44,56,69,90]
	>10 mL			[22]
Microfluidic-based Techniques	<1 mL	High	Parallelized chip design Continuous flow production Automated fluid handling High-throughput droplet generation	[30,43,44,45,54]
Hydrogel-Based Platforms	<1 mL	Moderate	Standardized hydrogel formulations Multi-well plate compatibility Automated gelation control Scalable precursor synthesis	[38,47,62,83,85,87,88]
Micromolding & Microwell Systems	<1 mL	Moderate	High-throughput mold production Standardized well geometries Automated cell seeding Parallel processing capability	[31,37,66,83]
Droplet & Particle Fabrication	<1 mL	High	Continuous flow production High-throughput droplet generation Automated particle collection Size control standardization	[24,43,45,75]
Photo-crosslinking/ Photo-polymerization	<1 mL	Moderate	Automated dispensing systems Controlled light exposure protocols Precursor solution standardization Multi-well plate compatibility	[57,59]
Lyophilization	<1 mL	High	Industrial freeze-drying equipment Batch processing in multi-well formats Precise parameter control Scalable biomaterial production	[46,61]
Templating	<1 mL	Moderate	Mass production of template materials Batch processing with molds Automated cell loading systems Standardized punching/cutting tools	[48,84]
Cell Sheet Engineering	<1 mL	Moderate to High	Stackable modular design Automated layering systems Microfluidic integration for vascularization In vivo assembly potential	[51,55]
Mechanical Fabrication	<1 mL	Low to Moderate	Process standardization Tool automation Material consistency control Batch processing adaptation	[27,60]
Plant-Based Constructs	<1 mL	Moderate to High	Abundant plant material sourcing Batch decellularization processing Standardized cleaning protocols Potential bio-ink development	[72,80]
Electrospinning	<1 mL	Moderate	Multi-jet electrospinning systems Continuous fiber collection Large-area deposition Industrial textile manufacturing adaptation	[42]
Surface Coating & Modification	<1 mL	Moderate to High	Automated coating systems Batch processing capability Precise coating parameter control Scalable biomaterial production	[65]
Bioreactor Systems	1–10 mL	High	Scalable reactor design Perfusion parameter optimization Standardized operation protocols Multi-unit parallel operation	[82]
Large-Volume Fabrication	>10 mL	High	Industrial-scale equipment adaptation Automated large-volume material handling Integrated perfusion and monitoring systems Automated quality assurance	[68]
Chemical Induction Systems	<1 mL	Moderate	Standardized chemical formulations Parallel culture systems Process automation potential Quality control protocols	[58]

**Table 8:** Summary of microenvironment criteria of 3D bioartificial liver constructs for implantation.

Category	Evaluation Methods/Markers	Key Functional Implications	Limitations	References
Vascularization	Endothelial identity: CD31/PECAM-1 immunostaining; 3D structure: confocal microscopy; in vivo remodeling: H&E staining	Enables oxygen/nutrient delivery, increases viable tissue thickness beyond diffusion limit, improves ALB, urea, CYP450 activity	Indicates advanced tissue maturity and spatial functional heterogeneity	[21,27,32,33,39,41,44,48,53,55,56,68,74,76,77,78,89]
Dynamic perfusion conditioning	Dynamic flow via microfluidic chips, perfusion bioreactors, tubular systems; functional readouts: ALB, urea, glucose consumption, CYP450; culture duration (7–30 days)	Sustains long-term viability and metabolic stability in thick constructs	Shear stress rarely quantified; flow often selected based on oxygen demand rather than physiological mechanics	[22,23,25,29,30,35,45,51,52,64,73,82]
Physiological stiffness	Elastic modulus (0.4–2.0 kPa); methods: AFM, rheometry, tensile/compressive testing, YAP/TAZ immunostaining	Maintains hepatocyte phenotype; suppresses fibrotic or dedifferentiated states; regulates YAP/TAZ signaling	Stiffness treated as static parameter; limited evaluation of temporal changes and implantation relevance	[42,50,57,60,62,70,79]
Polarity/biliary structure	Polarity markers: HNF4α, HNF1β, ZO-1, MRP2; biliary markers: CFTR, DPPIV, CK7/CK19; geometric guidance of bile ducts	Enables vectorial bile secretion and spatial organization essential for implantation	Mostly structural validation; functional bile transport and long-term stability rarely assessed	[26,54,58,63,67,87,88,90]
Metabolic & synthetic functions	Global markers: total bile acids (TBA), fibronectin production, ammonia metabolism; survival in liver failure models	Reflects integrated tissue-level hepatic performance and therapeutic relevance	Rarely assessed; often secondary endpoints without longitudinal profiling	[38,47,69,84,85]
Zonation	Oxygen/nutrient gradients via microfluidic or spheroid systems; differential CYP450 expression (heatmaps); OCR with computational modeling	Indicates advanced tissue maturity and spatial functional heterogeneity	Least standardized category; limited long-term and post-implantation validation	[28,31,37,66,91]

AFM: atomic force microscopy, ALB: albumin, CD31: cluster of differentiation 31, PECAM-1: platelet endothelial cell adhesion molecule-1, H&E: hematoxylin and eosin, CYP450: cytochrome P450 enzyme(s), TBA: total bile acids, OCR: oxygen consumption rate, HNF4α: hepatocyte nuclear factor 4 alpha, HNF1β: hepatocyte nuclear factor 1 beta, ZO-1: zonula occludens-1, MRP2: multidrug resistance–associated protein 2, CFTR: cystic fibrosis transmembrane conductance regulator, DPPIV: dipeptidyl peptidase-4, CK7/CK19: cytokeratin 7/cytokeratin 19, YAP/TAZ: Yes-associated protein/transcriptional coactivator with PDZ-binding motif.

**Table 9:** Summary of implantable artificial liver constructs evaluated in animal models.

Construct Category	Working Principle	Biomaterial	Cell Type (s)	Target Animal Models	Transplantation Periods	Advantages	Limitations	References
**Decellularized Liver Constructs**	Use native ECM from whole organs or lobes preserving liver-specific architecture and vascular channels.	Liver dECM (rat, porcine, human), sometimes combined with hydrogels (alginate, collagen).	Primary hepatocytes, hepatic progenitors, endothelial cells, occasionally MSCs.	Mice, rats, pigs.	1–30 days in rodents; up to 24 h in large-animal perfusion models; some survival studies 2–8 weeks.	Highly biomimetic; supports hepatocyte phenotype; allows potential vascular anastomosis.	Limited recellularization; perfusion failure; thrombosis risk; technically demanding.	[14,30,35,38,40,41,50,53,74,76,86,89]
**Hydrogel-Based Constructs (Non-bioprinted)**	Hydrogels encapsulate hepatocytes or HLCs; integration via host angiogenesis and diffusion.	Fibrin, PEGDA, PEGDA/HAMA, collagen I, gelatin, pullulan–dextran, cryogels, hybrid NiTi–hydrogel constructs.	Primary hepatocytes, HLCs, MSCs, ASCs, endothelial cells.	Mostly mice and rats; some SCID/NOD-SCID models.	1–4 weeks commonly; some chronic models 4–8 weeks; ALF rescue models 1–14 days.	Tunable mechanics; easy implantation; high cell retention; customizable microenvironment.	Diffusion limits; slower vascularization; limited thickness; shorter viability without vascular cues.	[27,31,34,43,44,46,48,51,59,60,62,69,72,75,78,80,85]
**3D-Bioprinted Liver Constructs**	Spatial deposition of hepatocytes, endothelial cells, and ECM-based bioinks; can include perfusable channels.	Collagen–chitosan, gelatin–alginate, dECM bioinks, vascular-channel bioinks.	Human hepatocytes, HLCs, iPSC-hepatocytes, MSC-derived hepatocytes, endothelial cells.	Mice and rats (orthotopic or mesenteric implantation).	1–4 weeks typical for functional evaluation; ALF rescue models 24–72 h for early endpoints.	High architectural control; supports vascularization; scalable; strong in vivo maturation.	Requires specialized equipment; necrosis risk in thick tissues; surgical complexity.	[47,50,64,68,79,81]
**Stem-Cell–Derived Hepatic Constructs**	ASCs, MSCs, or iPSC derivatives seeded in constructs differentiate into HLCs and exert paracrine regenerative effects.	Collagen I constructs, hydrogels, ECM hydrogels, composite constructs.	MSCs, ASCs, iPSC-derived hepatocytes, AHLCs, co-cultured immune or stromal cells.	SCID/NOD-SCID mice, rats with fibrosis or ALF.	2–6 weeks typical; some ALF models shorter (1–14 days).	Autologous potential; strong immunomodulation; regenerative cytokine secretion.	Lower maturity than primary hepatocytes; variable function; slow in vivo hepatic differentiation.	[35,44,47,72,75,78,79,80]
**Perfusable/Vascularized Bioengineered Devices**	Constructs contain vascular channels or microfluidic circuits connected to host vessels to provide continuous perfusion.	PEG-based hydrogels, 3D-printed vascular polymers, ECM-infused channels.	Primary hepatocytes, endothelial cells, stromal cells.	Rats (artery/vein anastomosis models), mice.	Acute perfusion 24–72 h; viability/function assessed 1–14 days.	Superior oxygenation; supports thicker tissues; enhanced hepatocyte viability and metabolic output.	Thrombosis risk; complex anastomosis; device hemocompatibility challenges.	[30,44,48,53,55,68,76,77,78]

## Data Availability

No new data were created or analyzed in this study. Data sharing is not applicable to this article.
